# A method to detect uniformity of road base course based on impact imaging technology

**DOI:** 10.1038/s41598-024-63941-9

**Published:** 2024-06-13

**Authors:** Xing-Guang Chen, Hao Luo, Shao-Kong Feng, Hai-Yang Wang

**Affiliations:** 1 College of Water Conservancy and Civil Engineering, South China Agricultural University, Guangzhou, 510640 China; 2 Guangdong Communication Planning & Design Institute Group Co., Ltd., Guangzhou, 510630 Guangdong China; 3 Ocean and Civil Engineering, Shanghai Jiao Tong University, Shanghai, 200240 China

**Keywords:** Impact imaging method, Pavement base course, Concealed disease, Detection method, Uniformity, Engineering, Materials science

## Abstract

The damage of road base course has the characteristics of strong concealment and difficulty in detecting. For this reason, the impact imaging method has been used for detection of road base course. This paper discussed systematically collection points setting, excitation mode and data processing method. Through the application in testing for highway pavement base before and after grouting maintenance, the results show that the method is simple and accurate. The detection results can be displayed in a two-dimensional image form and it is easy to be used in road maintenance. This method can be used to identify and locate the damages of the pavement base, to judge the uniformity of the pavement base structure. It can also be used to evaluate the effectiveness of internal damage after grouting repairing.

## Introduction

Cement stabilized bases with the outstanding advantage of using local materials, low cost, but high strength and good stability has been widely adopted in highways and municipal roads^1,2^. Nevertheless, with the continuous use of roads, particularly under heavy traffic loads, the cement-stabilized base course exhibits various shortcomings^3,4^.

These include susceptibility to cracking due to shrinkage, vulnerability to water damage, limited fatigue resistance, and sensitivity to overload, among others^1,5^. The damages to the base course of the road are located deep within, making them highly concealed and challenging to detect and differentiate through visual assessment at the initial stages. When the damages have become more deteriorative to be revealed, the pavement surface would be reflectively bottom-up destroyed already. The damages are so serious that they needs to be repaired in the excavation maintenance, which is more complicated with great expense of time and economic. Therefore, it is very important and significant to detect the damage of road bases accurately in the early stage^6^. It was a big challenge for traditional technologies.

Currently there are several non-destructive technologies to be used in detecting road base damage, like rebound method, ground penetrating radar, ultrasonic method and so on^7–9^. However, due to the complexity of road construction and environmental factors, the accuracy of these detection technologies may not meet expectations in practical projects. The rebound method is cost-effective and easy to use, but it has low accuracy and is greatly influenced by the road surface condition^10^. Ground-penetrating radar provides visual results in image form rather than data, heavily influenced by the subjectivity of researchers^11^. The ultrasonic detection method is a rapid and non-destructive testing technique with broad applications, but its ability to locate voids relies heavily on the operators' experience and knowledge^12^. A convenient and effective method that can assess the uniformity of the road base is essential for diagnosing disengagement, cavities, and leaks within the road.

Impact imaging method was used in geotechnical engineering originally, it was also adopted in the monitoring process of synchronous grouting filling for shield tunnel construction to determine the benchmark value of compactness. A method based on impact imaging technology is proposed in this paper to detect internal damage of road. Through the analysis of time variances in elastic wave reflections among monitoring points, discrepancies in waveform patterns along the monitoring line or area can be identified. These variations indicate differing levels of filling quality among monitoring points, facilitating the differentiation between approved and unapproved grouting zones.

This paper first briefly introduces the principle of impact imaging method at first, then major of utilization method and processing techniques in road base project are discussed in details. Finally, this technology has been employed in a real project to verify its availability and accuracy.

## Principle of impact imaging method

Impact imaging method is a non-destructive method based on elastic waves which could be generated when a temporary impact is applied on object structures^13,14^. The wave will be reflected strongly or transmitted for difference of wave impedance in different medium boundary or flaws position (like hollow, cavity and other damages)^15–17^. Impact imaging method is analyzing reflected waves and tracking the change to detect and judge the details inside the road^18^.

**Figure 1:**
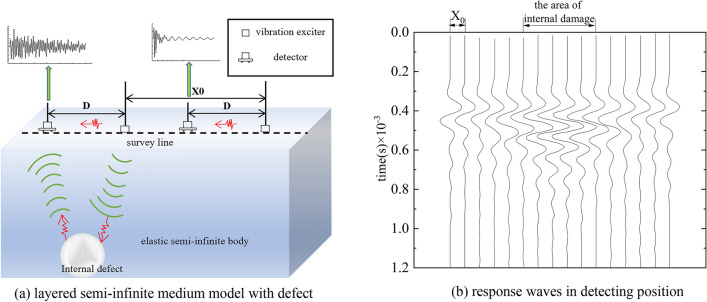
Principle of damage detection based on impact imaging method.

The road structure is simply considered as a layered semi-infinite medium model shown in Fig. 1a. There are some detectors installed on road surface to inspect internal damages. The internal defect and road surface can both be interpreted as strong deflection interface. Therefore, after vibration exciting the elastic wave will be generated and deflected several times on those two interfaces. The wave received by detector finally can be expressed as:

1 $$A\left(t\right)=\sum [w\left(t\right)\cdot r\left(t-n{T}_{0)}\right]=w(t)\cdot r(t)\begin{array}{c}=\text{1,2},3\dots n\end{array}$$

2 $$\begin{array}{c}r\left(t\right)=\left(R,-{R}^{2},{R}^{3},-{R}^{4}\dots \right)\end{array}$$

where $r(t)$ is reflection coefficient array, $R$ is reflection coefficient of internal defect, $T_{0}$ is reflection time of wave between two interface.

It shows that the elastic wave received by detector is convolution results of reflection coefficient and the wavelet. Consequently, if the wavelet of vibration source is known, the internal damage position and depth can be detected out through wave impedance difference of two interfaces which is obtained from reflection coefficient array by deconvolution operation. In actual detection, assuming that vibration exciters are installed in same line along survey direction at every ${X}_{o}$, the response wave signal will be received by detector sensor located at $D$. The changes in internal structures will be obviously revealed by arranging waveforms together shown in Fig. 1b. By analyzing waveform characteristics such as average amplitude and frequency, it is possible to infer the information of defect inside the road.

## Detecting strategy of road base damage based on impact imaging method

### Monitoring system

**Figure 2:**
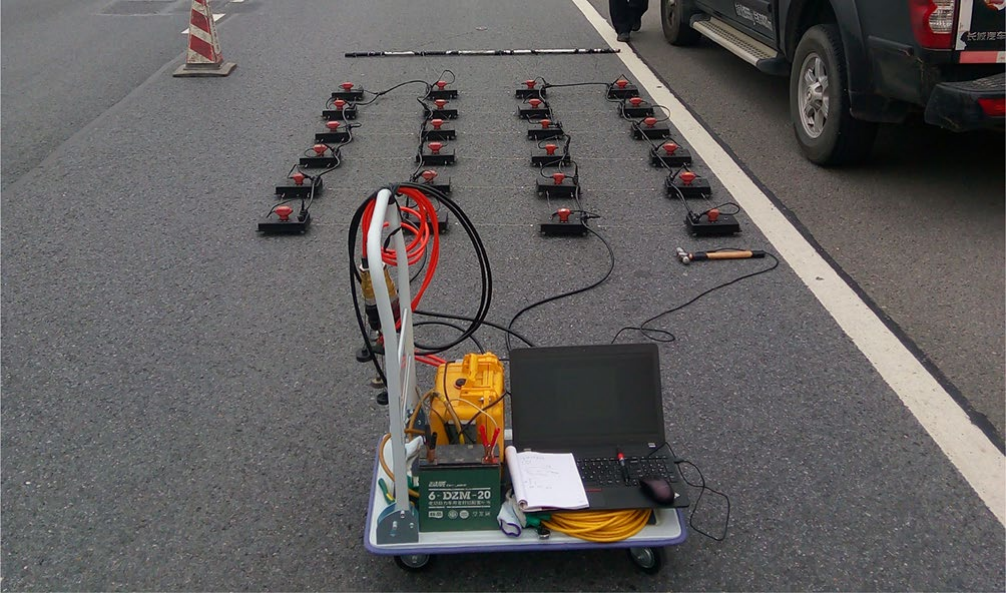
Monitoring system.

The detecting system includes an exciting device, an acquisition system, and a control system (as shown in Fig. 2). The exciting device is an impact hammer with a mass of 8 kg. The acquisition system produced by CGE (Chongqing) Geological Instrument Co., Ltd includes 24 sets of moving coil velocity transducers, each designed for vertical component (z-direction) velocity measurements with a natural frequency of 100 Hz. Additionally, the acquisition system utilizes a high precision seismograph, specifically a 24-channel Geode digital seismograph. This seismograph features 24 recording channels, 24-bit analog-to-digital conversion, a high cut-off frequency of 20,000 Hz, and a low cut-off frequency of 1.75 Hz.

### Scheme of detector arrangement and data acquisition

**Figure 3:**
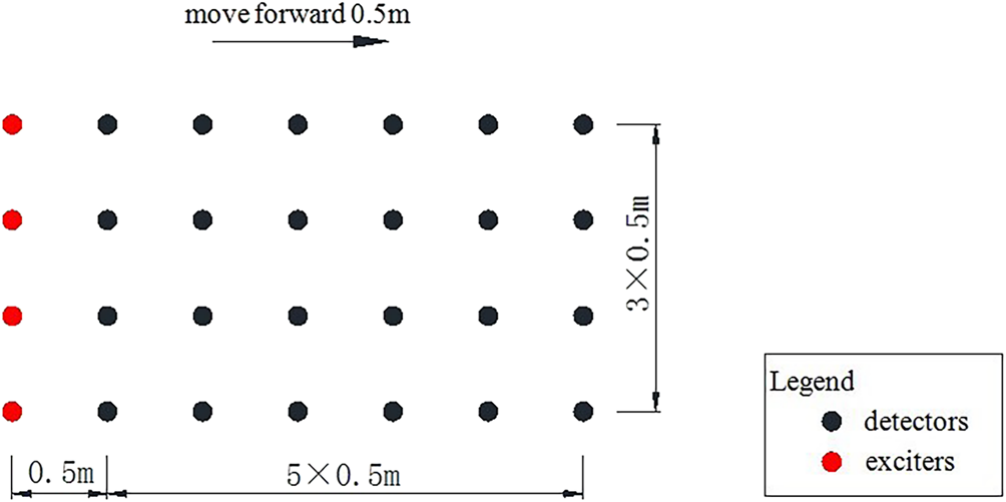
Arrangement of detectors and exciters.

In order to improve the accuracy of detection and reduce the influence of environmental noise, the detectors are installed as shown in Fig. 3.

Four survey lines are set up in the inspection lane. Twenty-four detectors are arranged in a 4 by 6 matrix configuration with a 0.5-m interval, identified as black points in Fig. 3. The 4 red points are vibration exciters which are 0.5 m behind detectors and excited one by one. 24 channels of data can be acquired at one excitation. Subsequently, the above process will be repeated after all detectors and exciters are move forward 0.5 m. In this method, data from 20 sensors in every 5 rows will be overlapped. Except first row and last 6 rows, all other detecting positions have 6 arrays of data, in each array, there are 4 response waveforms excited by 4 different exciters. Assuming that vibration waveform is same, the noise will be filtered through iterative computations by using 24 arrays data in each main position to get more accurate response waveforms.

### Method of data analysis

**Figure 4:**
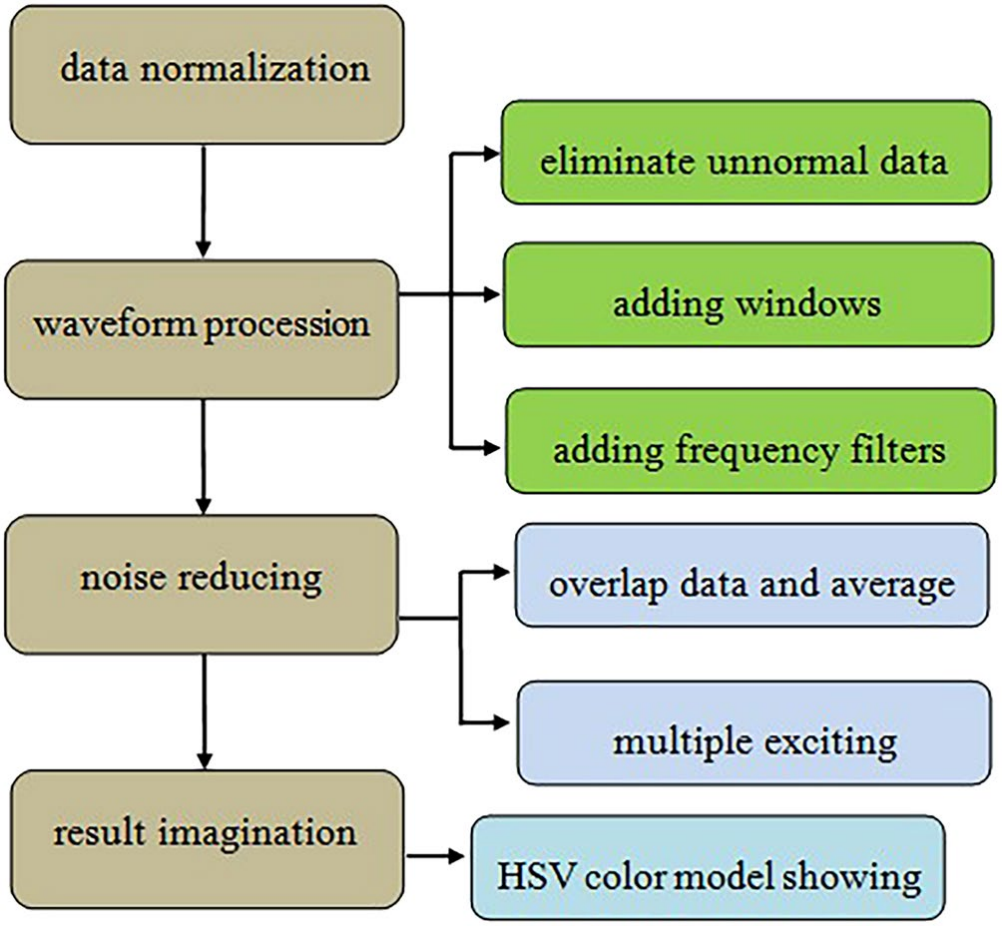
Flow chart of data analysis processing.

To obtain more useful information to better exhibit internal defect, the collected data have been processed through normalization, waveform procession, noise reducing and result imagination. The data analysis process is described clearly in Fig. 4.

Usually it will cost some time to complete the inspection for one section of road, and detection environment also changes from time to time, so it is difficult to keep exciting intensity constant all the time. The acquired data need to be normalized according to impact exciting vibration to reduce disturb from exciting intensity change.

Due to various accidental factors or unavoidable interference factors, there could be some abnormal data (less than 1%) in the array which will be pulled out and replaced by alternative data from interpolation method. If the time period of interference is different from the detected signal time, the time windows are employed to eliminate disturbed data out of windows. Meanwhile, frequency filters are designed to deal with the data which failed processing through above methods.

For one inspected point, the data from 2 to 4 times exciting will be overlapped and averaged to reduce noise, and detectors along same survey line could collect data from different exciting points to improve ratio of signal to noise. Although more redundant data and difficult of data processing will be increased by using multiple sampling and exciting point, it is available and significant to improve the reliability and accuracy of test results.

For better describe the detecting result, the response intensity distribution image based its absolute or relative value is used to show health information of all road segments. The hue saturation value (HSV) color model with blue–yellow–red is employed to express distribution of response intensity^19,20^. For the road with good condition, it shows blue–green in imaging, but in damage position it shows yellow–red because of strong response^21^.

### Implementation method

By utilizing the known speed of sound in materials (typically obtained through experiments or referenced data), we can calculate the wave propagation speed. By measuring the time taken for elastic waves, generated at the exciters, to reach 24 detectors after reflection, the approximate depth of defects can be determined.

In the field of non-destructive testing for road base damage, the thickness of road base course is generally less than 1 m, while the propagation speed of elastic waves can be as high as 5 km/s, resulting in very short travel times. At the same time, various waves generated during excitation, such as surface waves, direct longitudinal waves, reflected longitudinal waves, and converted waves, overlap, making it impossible to distinguish them through visual inspection. Therefore, higher precision data processing and analysis methods are required to accurately locate and assess the state of internal defects.

This project employs a data acquisition system developed for testing. This system consists of an acquisition instrument, a data collector, and a host, featuring lightweight, flexibility, ease of movement, high integration, and strong adaptability. Using Surfstar data processing software, high-precision processing of the reflected waves in both the time and frequency domains is conducted to obtain information on the size, shape, and location of medium defects.

## Practical validation

To practice the impact imaging method detecting uniformity of road base, the inspection project has been implemented on Guangzhou Airport Expressway which was constructed in 2002. It is the main road connecting Guangzhou New Baiyun International Airport and Guangzhou City. The daily average traffic volume is more than 200,000 natural trains.

The expressway adopts a pavement structure of 18 cm asphalt surface layer and 50 cm cement stabilized base layer. Over the years, hidden damages in the base layer have become more severe, aside from some structural issues like frost boiling and pumping. To improve maintenance efficiency and save maintenance time, the non-excavation with grouting method is adopted to repair those damage. The impact imaging technology is used before and after grouting to detect uniformity of road base to ensure the accuracy and reliability of maintenance process. 6 sections on expressway have been detected and repaired in the project, the forth lane in K16 + 9015 section is an example to show the results in the paper.

**Figure 5:**
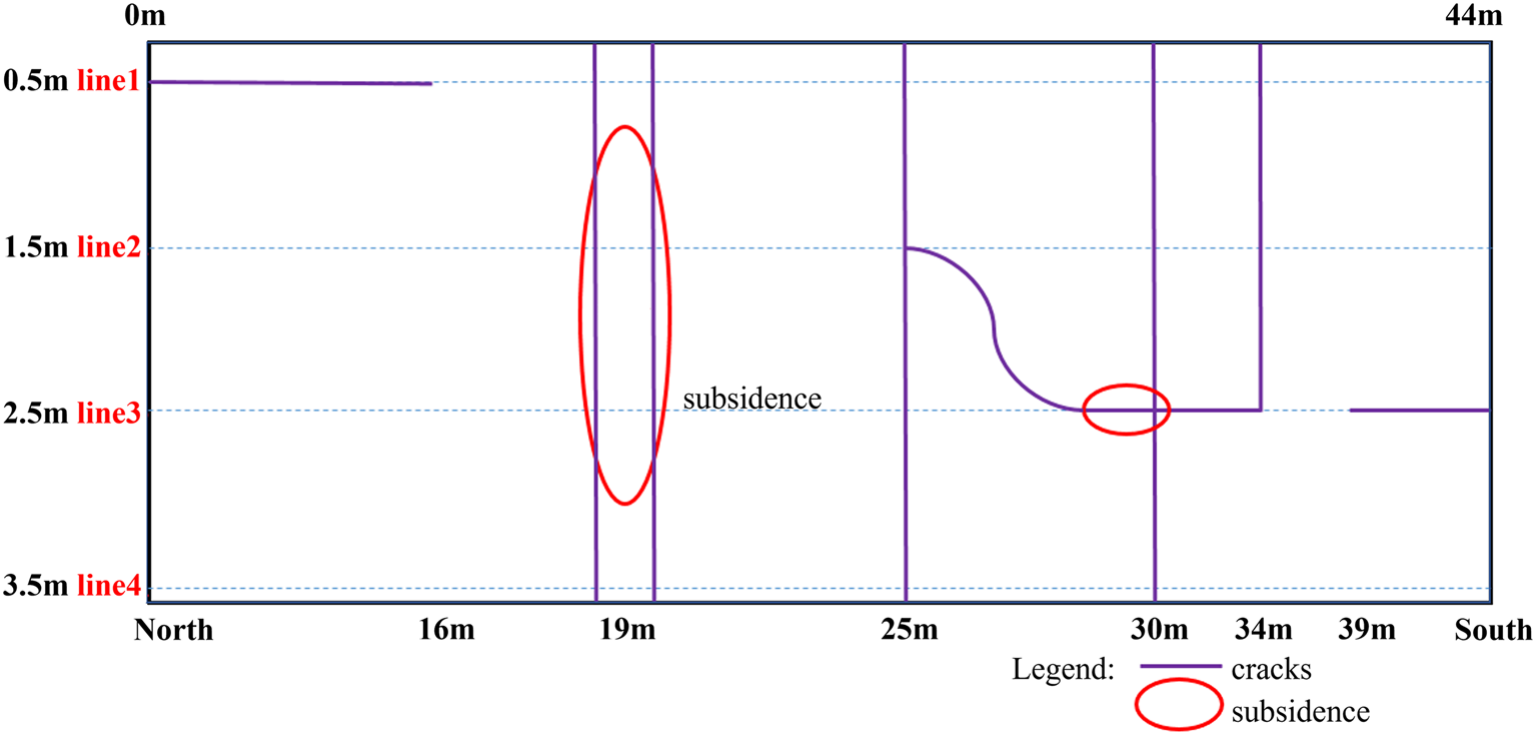
The condition of the pavement surface.

The basic condition of the road section is shown in Fig. 5, with longitudinal cracks at 1–16 m, 25–34 m, 39–44 m, transverse cracks at 19 m, 25 m,30 m, 34 m, and subsidence in 19–20 m, 29–30 m.

### Data analysis

The response intensity distribution images before and after grouting have been obtained through relative value of impact response amplitude by normalization and filtering. To make sure the agreement in expression, the response intensity from a section of road with good condition was employed in dimensionless procession for other detecting results, the updated relative value have been represented by chromaticity blue-green-yellow-red on plane graph.

**Figure 6:**
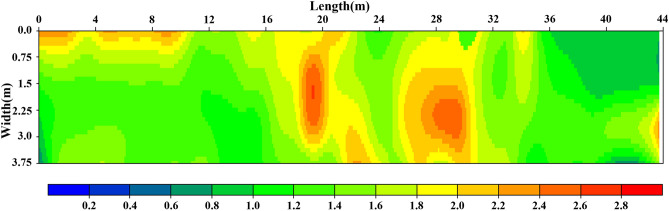
The response intensity distribution image before grouting.

Figure 6 shows the detecting results by using impact imaging method, it can be seen that there are serious cracks and road subsidence in the pavement in corresponding area, and the asphalt surface layer is seriously damaged. Comparing the description of practical condition in Fig. 5, it has the excellent agreements.

**Figure 7:**
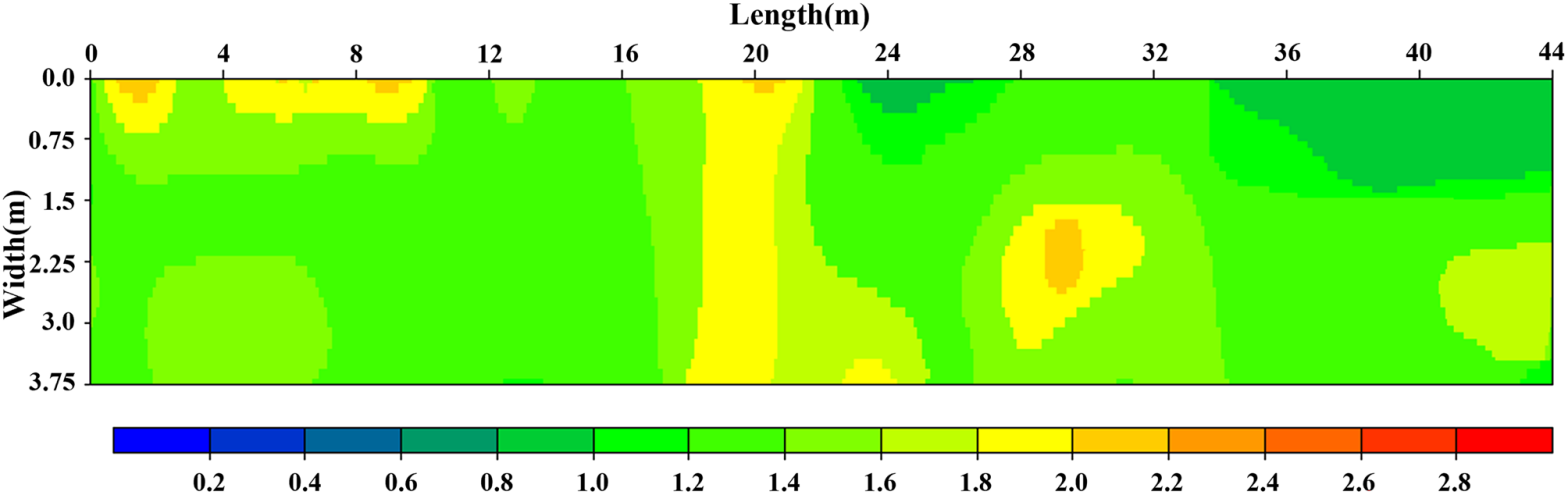
The response intensity distribution image after grouting.

The objective road had been repaired through grouting and checked the effect with impact imaging method as well shown in Fig. 7. The response intensity after grouting is obviously reduced, distribution image mainly coloured with green–blue shows that grouting has the good efficacy on helping road base layer getting high compactness. Although it still shows yellow–red in the range of 19–20 m and 29–30 m, the impact response intensity has been decreased significantly, indicating that the damages in those areas has improved significantly. There are still loose areas needing further treatment.

The images before and after grouting are all highly consistent with actual condition, which indicate that the impact imaging technology has high accuracy on detecting internal damage of road.

**Figure 8:**
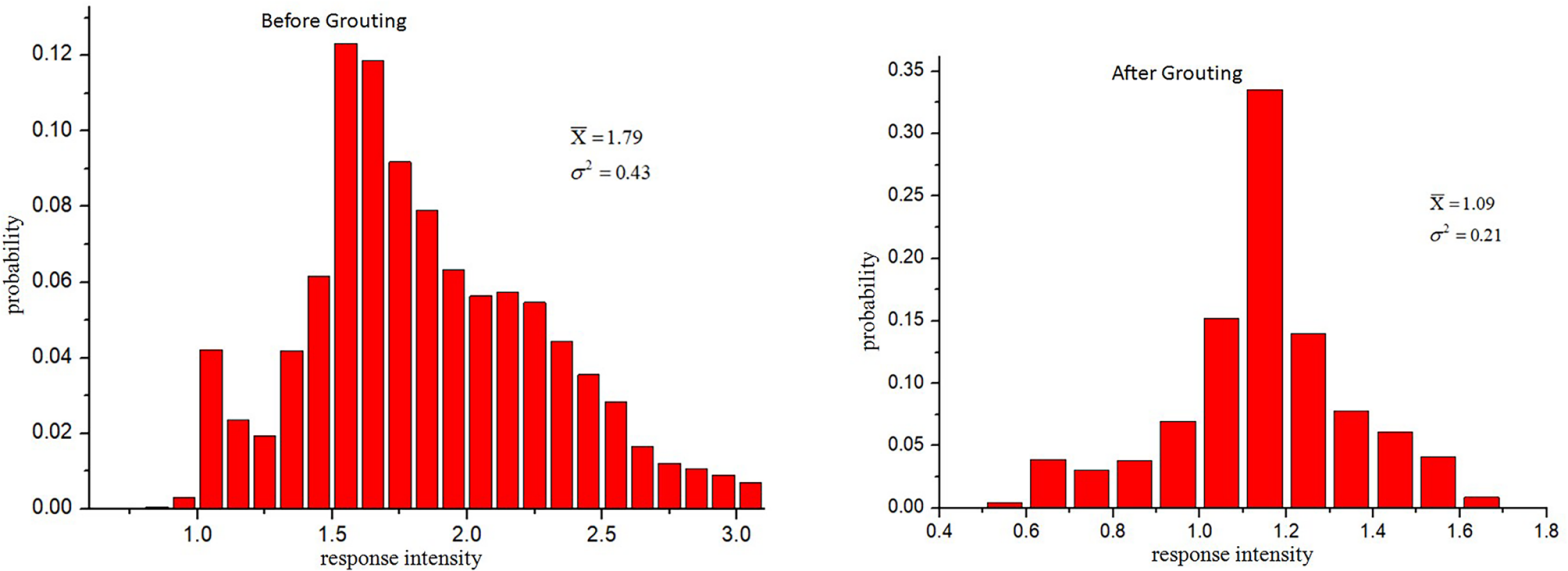
The probability distribution of the impact response intensity before and after grouting.

The probability distribution of the impact response intensity obtained before and after grouting are shown in Fig. 8. Before grouting, the mean value of impact response intensity of inspected section was 1.79, the variance was 0.43, and the maximum value was 3.1. After grouting, the mean, the variance and maximum value have become to 1.09, 0.21and 1.7 respectively. This indicates that the average strength of the repaired section of road after the grouting increased obviously, and the distribution of the impact response intensity is also more concentrated.

According to the calibration on-site, when the impact response intensity is less than 1.2, the probability of damage occurred in the road base layer is small. Therefore, it can be considered that the strength of the base structure is reliable and uniform when the impact response intensity is less than 1.2, which can be used as a basis for judging whether maintenance is required. Before grouting, the probability of the area with impact response intensity greater than 1.2 is 91.15%, and the probability is reduced to 18.97% after grouting, indicating that the structural uniformity and reliability of the base layer of the road section are significantly improved after grouting.

### Result validation

**Figure 9:**
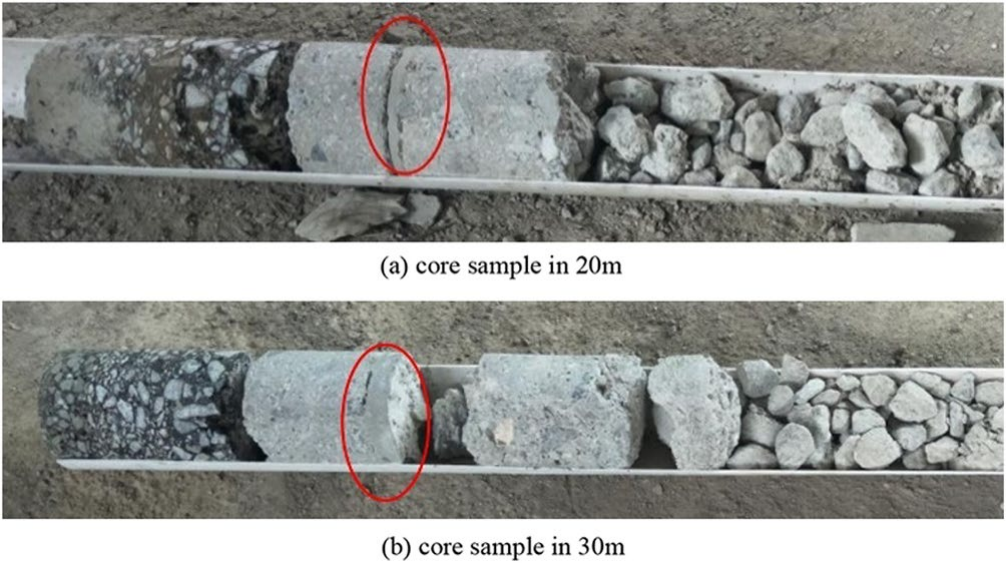
The core samples after grouting.

In order to verify the reliability of the detecting results, the road coring was implemented on inspecting section after grouting. Since the maximum depth of the core pulling device for pavement testing is 500 mm, which is smaller than the depth of the road base, the geological drilling rig was used to core. The core positions are located at 20 m and 30 m distance on inspected road section. The core samples are shown in Fig. 9.

From Fig. 9, it is obvious that there are some empties in road base layer before grouting, the grouted body has filled the cavity successfully, which also verifies the accuracy of the impact imaging technology.

## Conclusion

1. For addressing the issue of less accurate and precise traditional techniques in detecting internal damage of road base layers, a novel impact imaging method is introduced in the research paper. This method is proposed for identifying base layer damage, with a detailed design and discussion of the implementation scheme provided. Through verification in a real-world project, it was demonstrated that this method offers the benefits of high accuracy, ease of operation, visual output, and convenience in maintenance projects.
2. Using the impact imaging method, the uniformity of road base structure before and after grouting have all been compared with actual condition in the objective road section, the results show the excellent agreements, which signifies that impact imaging technique can be applied not just for identifying road base impairments, but also for assessing the efficacy of maintenance.
3. However, utilizing this approach to identify road base structure diseases poses certain challenges. Firstly, there is no special inspection equipment, the existing detection system with the disadvantage of moving slowly and manual excitation is difficult to satisfy the needs of large-scale inspection of highways and municipal roads. There are some safety risks in no-closing traffic road. Secondly, there is no standard indicator system to assess the effect, the test conclusions are greatly influenced by the experience and cognitive level of the operator.

In the next phase of future system research, a dedicated detection device will be developed for impact imaging method to enable features such as on-vehicle motion and mechanical impact detection, enhancing the method's effectiveness and reducing reliance on human resources. This will expand the application of impact imaging method to further road infrastructure maintenance projects, establishing a comprehensive index system, ultimately leading to more accurate and efficient test results while minimizing external factors' impact.

## Data Availability

The data that support the findings of this study are available from the corresponding author, W.H., upon reasonable request.
